# 2743. Pre-Transplant Human T-Lymphotropic Virus Screening In Allogeneic Hematopoietic Stem Cell Transplant Recipients At A Large Metropolitan Tertiary Referral Centre In Melbourne, Australia

**DOI:** 10.1093/ofid/ofad500.2354

**Published:** 2023-11-27

**Authors:** Gabriel Paykin, Janine Trevillyan, Andrew Gador-Whyte, Sarah E Garner, Peter Shuttleworth, Jason Trubiano, Olivia Smibert

**Affiliations:** Austin Health, Melbourne, Victoria, Australia; Austin Health, Melbourne, Victoria, Australia; Austin Health, Melbourne, Victoria, Australia; Austin Health, Melbourne, Victoria, Australia; Austin Health, Melbourne, Victoria, Australia; Austin Health, Melbourne, Victoria, Australia; Austin Health, Melbourne, Victoria, Australia

## Abstract

**Background:**

Pre-transplant infectious diseases screening is an essential component of the pre-transplant workup. Many centres that offer allogeneic hematopoietic stem cell transplantation (HSCT) routinely screen for human T-lymphotropic virus (HTLV) in all recipients in the pre-transplant setting. The aim of this single centre retrospective chart review was to assess pre-transplant HTLV screening rates in allogeneic HSCT recipients at our institution in Melbourne, Australia and determine the prevalence of HTLV in this cohort.

**Methods:**

Patients who received an allogeneic HSCT between 1st January 2018 and 31st December 2022 were identified from an institutional database. Patients who received a second HSCT during the study period were excluded from this analysis. Demographic and clinical data were extracted from electronic medical records. Descriptive analysis of the patient cohort was conducted. Data on the number and outcome of HTLV screening tests performed in this cohort between 1st January 2017 and 31st December 2022 were collated in order to determine the rate of pre-transplant HTLV screening and prevalence of HTLV in this cohort.

**Results:**

Over the study period 116 patients received an allogeneic HSCT at our institution. Of these, 110 were included in this analysis. 67 (60.9%) were male and 43 (39.1%) were female. The mean age at time of transplant (D0) was 51.0 years. Eight (7.3%) patients received haploidentical transplants, 20 (18.2%) received matched unrelated donor transplants, 44 (40.0%) received sibling transplants and 38 (34.5%) received unrelated donor transplants. 158 pre-transplant HTLV screening tests were performed on included patients over the study period. 75 (68.2%) patients had one pre-transplant HTLV test, 25 (22.7%) had two pre-transplant HTLV tests, seven (6.4%) had three pre-transplant HTLV tests and three (2.7%) had four pre-transplant HTLV tests. All HTLV tests included in this analysis were non-reactive on enzyme immunoassay screening.

**Conclusion:**

All patients included in this study had at least one pre-transplant HTLV screening test over the study period. There were no cases of HTLV detected in this cohort. Further research is required on the utility and cost effectiveness of routine pre-transplant HTLV screening in transplant recipients in non-endemic areas.

**Disclosures:**

**All Authors**: No reported disclosures

